# The knowledge, attitudes and practices of doctors regarding antibiotic resistance at a tertiary care institution in the Caribbean

**DOI:** 10.1186/s13756-018-0315-3

**Published:** 2018-02-15

**Authors:** Alison Nicholson, Ingrid Tennant, Livingston White, Camille-Ann Thoms-Rodriguez, Loraine Cook, Stephen Johnson, Tamara Thompson, Jasper Barnett, Lundie Richards

**Affiliations:** 1 Department of Microbiology, The University of the West Indies (UWI), Kingston, Jamaica; 2 Department of Surgery, Radiology, Anaesthesia and Intensive Care, UWI, Kingston, Jamaica; 3 UWI CARIMAC, Kingston, Jamaica; 4 School of Education, UWI, Kingston, Jamaica; 5 Department of Government, UWI, Kingston, Jamaica; 6 Department of Medicine, UWI, Kingston, Jamaica; 7 Ministry of Health, Kingston, Jamaica

**Keywords:** Antibiotic, Prescribing, Practices, Resistance, Knowledge, Attitudes

## Abstract

**Background:**

Antibiotic resistance (ABR) is a serious threat that requires coordinated global intervention to prevent its spread. There is limited data from the English-speaking Caribbean.

**Methods:**

As part of a national programme to address antibiotic resistance in Jamaica, a survey of the knowledge, attitudes and antibiotic prescribing practices of Jamaican physicians was conducted using a 32-item self-administered questionnaire.

**Results:**

Of the eight hundred physicians targeted, 87% responded. The majority thought the problem of resistance very important globally (82%), less nationally (73%) and even less (53%) in personal practices. Hospital physicians were more likely to consider antibiotic resistance important in their practice compared to those in outpatient practice or both (*p* < 0.001). Composite knowledge scores were generated and considered good if scored > 80%, average if 60–79% and poor if < 60%. Most had good knowledge of factors preventing resistance (83%) and resistance inducing potential of specific antibiotics (59%), but only average knowledge of factors contributing to resistance (57%). Knowledge of preventative factors was highest in females (*p* = 0.004), those with postgraduate training (*p* = 0.001) and those > four years post graduation (*p* = 0.03). Empiric therapy was often directed by international guidelines and cultures were not routinely done. Limited laboratory and human resources were identified as challenges.

**Conclusion:**

Physicians in this study were aware of the problem of ABR, but downplayed its significance nationally and personally. These results will guide a national antibiotic stewardship programme.

## Background

Antibiotic resistance (ABR) has emerged as a significant threat to the quality of health care in the twenty-first century [1]. This, combined with decreased production of new antimicrobial agents, has precipitated a global crisis with the emergence of bacteria resistant to most antibiotics [2–5].

The inability of previously effective antibiotics to treat common bacterial infections has far reaching consequences [1]. The success of many modern advancements in medicine is predicated on the availability and efficacy of antibiotics. These include surgery using prostheses, organ transplantation and chemotherapy [4]. The effects of ABR extend beyond increased mortality (63,000/year in the US and 25,000/year in the EU) [6] and morbidity to include increased hospital stay and costs. In the USA, ABR has been estimated to cost over 55 billion dollars per annum [7]. In Europe, estimated costs are at least €1.5 billion per year [6]. However, the real costs are higher when the impact of ABR on the entire health care system and national productivity is taken into account.

Of the over 20 countries in the English speaking Caribbean, data on antibiotic resistance are limited largely to reports from Jamaica, Trinidad and Tobago and Barbados [8–13]. This is concerning, as the Caribbean is a popular destination especially for North Americans and Europeans and this would have implications for the types of multiple drug resistant organisms (MDROs) present. A multiple drug resistant organism is defined as one that is resistant to one or more drugs in three or more drug classes [14]. In Jamaica, globally recognized “problem organisms” that have been identified include Methicillin resistant *Staphylococcus aureus* (MRSA), extended spectrum betalactamases (ESBL) *Klebsiella pneumoniae, Escherichia coli* and *Enterobacter sp.*, New Delhi metallo-beta-lactamase (NDM-1) *Klebsiella pneumoniae,* carbapenem resistant *Pseudomonas aeruginosa* and *Acinetobacter spp.* as well as Vancomycin resistant Enterococcus (VRE) [13, 15–17]. A 2009 study in a university-affiliated hospital in Kingston, Jamaica showed that resistance among Gram-positive organisms was much lower than among Gram-negative organisms [16].

Antimicrobial abuse is an important cause of ABR and is a compelling target for attention. “Antimicrobial abuse” is an umbrella term for a wide range of breaches including overuse, inappropriate choice, incorrect dosage, incorrect duration of therapy, incorrect dosing interval and suboptimal route of delivery [4, 18].

A single-centre study from Jamaica showed that although physicians were aware of ABR and contributing factors, this did not influence their prescribing practices [19]. With a desire to slow the emergence of antimicrobial resistance by determining the strategies necessary to improve prescribing practices among Jamaican physicians, a much larger all island multicentre study was carried out.

## Methods

### Aim and study design

A cross-sectional study was performed to identify the knowledge, attitudes and practices of Jamaican physicians towards antibiotic resistance and antibiotic prescribing practices. This information will help in the development of national antibiotic guidelines and workshops for healthcare workers. A problem with such strong global significance requires every country to define its challenges and make its voice heard.

### Study sampling and study instrument

This study was conducted across Jamaica, the third largest Caribbean island, 11,424 km^2^, with a population of approximately 2.8 million [20, 21]. Approximately 4000 public and private doctors were identified as currently providing medical care from national registration records (Jamaican Medical Council). We targeted 20% or 800 doctors. With this sample size, we calculated that we would have a margin of error of 4% and a 99% confidence level, assuming a conservative response distribution of 50%. We recruited through medical conferences, hospital meetings and visits to private practices across the four Jamaican health regions- South, Southeast, Northeast and West. Physician registration with the local medical council requires regular attendance at conferences or meetings to obtain continuing medical education credits (CMEs). In addition, we did not target microbiological conferences or those on antibiotics or resistance, but included a wide cross-section of meetings. Therefore, we do not believe that our sample was biased towards physicians with better knowledge of ABR. Data collection was done between October 2014 and September 2015.

The instrument used was a 32-item self-administered questionnaire comprising questions related to demographics (eight questions), knowledge (six questions), attitudes (five questions) and practices associated with antibiotic use (13 questions). Ethical approval was obtained from the Faculty of Medical Sciences, UWI (ECP 183 13/14) and Ministry of Health (MOH) Ethics Committees (2014/26) and informed consent was obtained from each participant.

### Data analysis

Data were analysed using Statistical Analysis in the Social Sciences (SPSS) version 19 and correlations assessed using the Chi-square test. A *p*-value of less than 0.05 was considered statistically significant.

Physicians’ knowledge of factors contributing to development of ABR and factors useful in containing resistance were examined. Each factor was analyzed on a scale of 0 to 3 (0 = don’t know, 1 = minimally important, 2 = moderately important and 3 = very important) and showed how the respondents ranked their importance. Knowledge of antibiotics more prone to inducing resistance was also assessed, and each was scored between 0 and 2 (0 = don’t know, 1 = less likely, 2 = more likely).

Composite knowledge scores were also calculated. The first score “*Factors contributing to development of ABR*”, represents a computation of 9 variables, each with 4 categories scored from 0 to 3 as follows: 0 = Don’t Know, 1 = Minimally Important, 2 = Moderately Important, 3 = Very Important. To ensure the variables were compatible with parametric analysis, the scale variable was converted to an ordinal variable; created by recoding into the following categories (9 to15 = Poor knowledge; 16 to 21 = Average knowledge, 22 to 27 = Good knowledge). This score was then cross-tabulated against physician variables to determine significant predictors.

The second score “*Factors useful in containing resistance*” was created from 8 variables, each with four categories (0 = Don’t Know, 1 = Not Useful, 2 = May be useful, 3 = Useful). This scale was recoded into three categories (Poor knowledge = 8–12; Average knowledge = 13–16; Good knowledge = 17–24).

The third score “*Antibiotics more prone to inducing resistance*” (which refers to the potential of an antibiotic to cause the emergence of resistance over time [22]) was created from 7 variables, each with three categories (0 = Don’t Know, 1 = Less likely, 2 = More likely). The overall score was calculated based on the number of correct responses, with a maximum of 14 points. This scale was recoded into three categories; (Poor knowledge = 0–4; Average knowledge = 5–9; Good knowledge = 10–14).

Reliability analyses using Cronbach alpha were done to determine the level of internal consistency among the items comprising the individual scales. Results indicated high Cronbach’s alpha scores of 0.749 to 0.859 for most of the scales. Only “*Factors contributing to development of ABR*” had a lower Cronbach’s alpha of 0.631, which is still acceptable [23].

## Results

### Respondent demographics

A total of 695 physicians completed the questionnaire, a response rate of 87%, with 51% female. The majority stated that their practice was mainly hospital based (60%) and 51% had postgraduate training. About a third (32%) were less than four years post registration and 33% were residents. (Table 1) The Southeast region accounted for 62% of the respondents, the Western region 22%, the Northeast region 5% and the Southern region 11%. This corresponded fairly well with data from the Ministry of Health, which indicated the following distribution of physicians: Southeast region 55%, Western region 20%, Northeast 15% and South 10% (J Barnett, pers. comm.).

**Table 1:** Physician Demographics

	Category	Frequency	Percent
Gender	Male	338	49%
	Female	348	51%
Current Practice	Mainly hospital based	387	60%
	Mainly outpatient or clinic based	158	25%
	An equal proportion of both	97	15%
	Total	642	100%
Position	Intern/SHO	118	17%
	Intern/resident	186	27%
	Senior/chief resident	43	6%
	Consultant	156	22%
	Private GP	120	17%
	Specialist private practice	38	5%
	Government clinic medical officer	34	5%
	Total	695	100%
Postgraduate Training	Yes	338	51%
	No	328	49%
	Total	666	100%
Specialty	Anaesthesia/intensive care	56	17
	Internal medicine	64	19
	Surgical specialty	102	31
	Paediatrics	27	8
	Obstetrics and gynaecology	24	7
	Accident and emergency	13	4
	Family medicine	42	13
	Clinical microbiologist	6	2
Years Post Registration	Total	334	100%
	0–4 years	218	32%
	5–9 years	130	19%
	10–14 years	87	13%
	15–19 years	61	9%
	>_ 20 years	176	26%
	Total	672	100%

N.B. Total sample size for this study is 695. Differences in totals for each subsection occur because of missing values

### Opinion on the magnitude of the problem

Most of the respondents (82%) felt the global problem of ABR was very important, but less considered the national problem very important (73%). In their personal practice, however, only 53% assessed resistance as being very important; and 15% did not think it was important at all. Hospital physicians were more likely to consider ABR very important in their practice than outpatient-based, or physicians who practiced in both areas (65% vs. 39% and 35% respectively, *p* < 0.001).

### Knowledge

#### Factors contributing to resistance

Widespread use of antibiotics was thought to be most important, with a mean score of 2.79 ± 0.48 followed by overuse of broad-spectrum antibiotics (2.74 ± 0.55) and inappropriate use of antibiotics (2.61 ± 0.60). Inadequate hand washing (1.47 ± 0.95) and use of antibiotics in the livestock industry (1.41 ± 1.03) were considered least important. (Table 2).

**Table 2:** Knowledge of Factors Contributing to Antibiotic Resistance

	Mean	SD
Widespread use of antibiotics	2.79	.48
Overuse of broad spectrum antibiotics	2.74	.55
Inappropriate use of antibiotic therapy	2.61	.60
Inappropriate initial choice of antibiotics	2.58	.62
Lack of guidelines on antibiotic usage	2.17	.81
Patients’ demand for antibiotics	1.90	.80
Role of pharmaceutical companies in promoting the use of antibiotics	1.75	.79
Inadequate hand washing	1.47	.95
Use of antibiotics in the livestock industry	1.41	1.03

Based on their overall score, the majority (57.4%) had average knowledge, 29.2% had good knowledge and 13.5% had poor knowledge of factors contributing to ABR. Higher knowledge of these factors was positively correlated to female sex (*p* = 0.004), having postgraduate training (*p* = 0.001) and > four years post graduation (*p* = 0.03).

#### Knowledge of preventing or containing antibiotic resistance

Physician education programmes (2.67 ± 0.66) and access to timely laboratory reports (2.67 ± 0.67) were both considered most important and antibiotic restriction (2.04 ± 0.80) and cycling (1.97 ± 0.92) least important (Table 3).

**Table 3:** Knowledge of Preventing or Containing Antibiotic Resistance

	Mean	SD
Ongoing physician educational programmes	2.67	.659
Access to timely lab reports	2.67	.674
Development of national antibiotic guidelines	2.63	.684
Access to microbiology consultations	2.58	.694
Development of better diagnostic tests to differentiate bacterial from viral infection	2.53	.745
Public educational programmes	2.44	.753
Antibiotic restriction: requiring a countersignature by a consultant or microbiologist	2.04	.800
Antibiotic cycling: switching routine use from one class to another at a regular intervals	1.97	.920

The overall score for knowledge of factors to contain resistance showed the majority of respondents had good knowledge (83%), 9.5% had average knowledge and 7.5% poor. A good score was positively correlated to type of current practice (*p* = 0.04), with the highest scores in physicians whose practice was mainly hospital based.

#### Antibiotics more prone to inducing resistance

Respondents incorrectly thought that amoxicillin-clavulanic acid had the greatest potential to induce resistance (1.51 ± 0.7), but correctly identified ceftriaxone (1.31 ± 0.75) and ciprofloxacin (1.23 ± 0.78) as inducers of resistance. Antibiotics thought to have the least potential to induce resistance were amikacin (0.94 ± 0.7) and the carbapenems (0.98 ± 0.7). (Table 4).

**Table 4:** Knowledge of Inducing Antibiotic Resistance

	Mean	SD
Amoxicillin-clavulanic acid	1.51	.70
Ceftriaxone	1.31	.75
Ciprofloxacin	1.23	.78
Ceftazidime	1.13	.74
Piperacillin-tazobactam	1.01	.70
Meropenem/Imipenem	.98	.70
Amikacin	.94	.65

(0 = don’t know, 1 = less likely, 2 = more likely)

Most (58.7%) had good knowledge, 25% had average knowledge and the remainder’s knowledge was poor (16%). This score was correlated to type of practice (*p* = 0.005); years post graduation (*p* = 0.018) and postgraduate training (*p* = 0.013). Physicians who were primarily hospital-based, who had been in practice between 10 and 14 years or had postgraduate training had the highest scores.

#### Optimal duration of therapy

Most physicians would treat an uncomplicated urinary tract infection for 7–10 days (43%), 30% would treat for five days and 24% for less than five days. For a group A streptococcus pharyngitis, 64% would treat for seven to ten days, 19% for five days and 12% for 14 days. A community-acquired pneumonia would be treated for 7–10 days by the majority (75%), and five days (13%) and 14 days (8%) by the remainder. A *Staphylococcus aureus* bacteremia would be prescribed 14 days of antibiotics by 48% of respondents, seven to ten days by 36% and > 14 days by 12%.

#### Complications related to antibiotic resistance

Respondents felt that increased costs related to care (73%) and prolonged hospital stay (66%) were the most frequent complications of ABR. Death and organ failure (33% respectively) were thought to be less common.

### Attitude

#### Opinion on restriction of antibiotics

The antibiotics considered suitable for a hospital-restricted list (multiple antibiotics could be chosen) included vancomycin (81%), carbapenems (79%) and piperacillin/tazobactam (65%). Most did not think that ciprofloxacin (27%), ceftazidime (28%) or ceftriaxone (14%) should be restricted. 36% of respondents felt the current level of restriction should be increased, 25% felt they should be maintained and 4% advocated a decrease.

### Practice

#### Personal experience with MDROs

Physicians identified MRSA (65% of respondents), multiresistant *Pseudomonas aeruginosa* and ESBL *Eschericha coli* (35% each), ESBL *Klebsiella pneumoniae* (28%), penicillin resistant *Streptococcus pneumoniae* (PRSP, 27%) and Vancomycin Resistant Enterococcus (VRE) (19%) within their personal practices. Hospital physicians and those with both a hospital and outpatient-based practice consistently reported higher personal experience with MDROs, except for PRSP. Hospital physicians reported less experience with PRSP (22%) than outpatient-based (30%) and those with a combined practice (34%, *p* = 0.03). 75% hospital-based, 72% of both and 41% outpatient-based physicians had experience with MRSA (*p* < 0.001).

#### Choice of antibiotics

The factors that commonly affected antibiotic choice included severity (91%) and site (90%) of infection; patient factors such as renal disease, immunocompromise and allergy (84%); availability of antibiotics (84%) and cost (58%). Knowledge of patterns of ABR was important for 54%. Only 5% of respondents admitted to being influenced by requests from the patient or their family and 2% to pressure from pharmaceutical companies.

For a community-acquired pneumonia, the most common initial empiric choices were amoxicillin-clavulanic acid (77%) and erythromycin (38%). For a hospital acquired pneumonia, ceftriaxone (38%), ceftazidime (33%) and piperacillin/tazobactam (31%) were the most popular choices. Trimethoprim-sulfamethoxazole (52%), amoxicillin-clavulanic acid (44%) and ciprofloxacin (41%) were commonly used for uncomplicated urinary tract infections. (Table 5) Physicians were allowed to choose more than one antibiotic for each type of infection, and so totals are greater than 100%.

**Table 5:** Choices of Empiric Antibiotics

	Community acquired pneumonia	Hospital acquired pneumonia	Uncomplicated urinary tract infection	Overall
Amoxicillin-clavulanic acid	77%	21%	44%	53%
Erythromycin	38%	11%	3%	14%
Ceftriaxone	21%	38%	6%	22%
Cefuroxime	15%	16%	10%	14%
Ciprofloxacillin	7%	13%	41%	20%
Bactrim	7%	5%	52%	18%
Ceftazidime	7%	33%	3%	13%
Metronidazole	6%	8%	5%	13%
Piperacillin/tazobactam	6%	31%	3%	11%
Meropenem	4%	12%	2%	5%
Amikacin	3%	9%	4%	5%

Forty-three percent (43%) of respondents frequently take cultures prior to starting a course of antibiotics, 47% occasionally and only 7% always take cultures. Frequently taking cultures was more common amongst hospital physicians (53%) than outpatient-based (24%) or those who had a combined practice (44%, *p* < 0.001). Most (53%) are able to get results within four to seven days, and 16% in less than three days. However, 27% complained that results would take over 1 week. 41% of respondents felt that their initial choice was correct based on laboratory reports “somewhat often” and 31% “sometimes” 5% had never checked.

#### Responses to culture reports

Physicians were asked what their response would be to a culture report indicating the organisms isolated were resistant to the empiric antibiotic/s in a patient responding clinically. The majority (54%) would change to antibiotics indicated by the report, 31% would continue the present antibiotics, and 16% would add one of the susceptible antibiotics indicated by the report. When asked what their response would be to a culture report showing an isolate sensitive to current antibiotics, but also to a narrower-spectrum antibiotic in a patient who is responding clinically, only 21% would de-escalate to the narrow-spectrum therapy.

#### Physicians’ treatment decisions when antibiotics are not indicated

When asked about their practice if they thought antibiotics were not indicated, 92% would explain to their patients why they were not needed, 88% would guide them on seeking follow-up care if symptoms do not improve, 84% would provide medications for relief of symptoms and 71% would educate on the harm of taking unnecessary antibiotics. Only 6% admitted to prescribing antibiotics if the patient demanded it. However, when further asked if they had ever prescribed antibiotics only because of patients’ insistence, 21% (12% hospital based, 31% outpatient based) admitted to doing that.

#### Additional training

Only 34% of respondents thought that their knowledge of antibiotics and ABR was good or very good; the majority (53%) felt it was average and this was the same for both hospital and outpatient-based physicians. The majority of respondents (86%) thought they needed a refresher course in ABR and prescription. Just over half of the respondents (52%) had further training in the use of antibiotics post graduation, either through a seminar (86%) or a formal course (14%).

The majority of physicians (74%) turned to the Internet when they needed further information on infection management. Other sources included consultation with local experts (54%), other colleagues (43%), national guidelines (41%) and textbooks (40%). Only 35% would consult institutional guidelines.

## Discussion

This is the first national survey of Jamaican doctors regarding their knowledge and opinions on antibiotic resistance and their prescribing practices. Physicians from all health regions were included from a wide range of disciplines and clinical experience.

### Knowledge and attitudes

Eight out of 10 doctors (82%) recognized the magnitude of ABR globally, but less were convinced of this locally (73%) and even less so in their personal practice (53%). The danger of this finding is that physicians could become detached from the problem, making them less likely to try to contain it. ABR is more common in the hospital setting and this was reflected in the responses of the hospital doctors. The authors have observed that the prohibitive cost and delay in retrieving microbiology reports in some areas have adversely affected the Jamaican physicians’ perceptions of the value of obtaining routine cultures. This could explain why only 7% of physicians always take cultures for a suspected infection. Hence, there is little reminder of prevailing resistance patterns.

Most physicians appreciated the influence of widespread use, inappropriate choices and overuse of broad-spectrum antibiotics as drivers of ABR [4, 18]. However a significant number failed to recognize important drivers of resistance such as inadequate hand hygiene and antibiotic usage in the livestock industry [4, 5]. When the objective assessment of their overall knowledge of ABR was compared to their own personal assessment, there was good correlation with 57% receiving an average score objectively, and 54% perceiving their knowledge as average.

Ongoing educational programmes, the development of national antibiotic guidelines, access to microbiology consultation, as well as improved laboratory services were thought to be most useful in containing ABR. There are only two medical microbiologists and one infectious disease specialist for the public health sector across the entire island. This needs to be urgently addressed as part of a national programme. Access to timely laboratory reports continues to be a challenge in this resource limited setting, where there are only few laboratories with limited diagnostic capacity, often using manual systems. Although this problem would appear to be a local one, its potential impact could be global because of Jamaica’s position as a popular destination. The role of inadequate rapid point of care (POC) tests to differentiate between viral and bacterial infections was also clearly appreciated by the respondents as being an important factor to contain resistance. POC tests are those that are performed at the bedside and generate convenient and rapid results [24]. These would quickly differentiate between viral and bacterial infections such as Group A Streptococcus and urinary Streptococcus pneumoniae. Unfortunately there are only a few rapid POC tests and this is a problem internationally [25]. This is particularly highlighted by upper respiratory tract infections which are common worldwide and are often overtreated by antibiotics in the absence of rapid POCs [26]. Factors such as antibiotic cycling and restriction, that would confine the physicians’ prescribing practice, were not perceived to be as important. This was confirmed by the fact that only just over one third (36%) thought that current antibiotic restriction policies should be increased.

Just over half of the physicians (59%) surveyed understood that some antibiotics are more prone to inducing resistance than others. As expected, physicians in the hospital setting had better knowledge, but there is room for more education across the board, such as seminars, workshops, conferences, newsletters and official antibiotic guidelines. Although the third generation cephalosporins and fluoroquinolones have a high propensity towards resistance induction [27] they remain some of the more prescribed antibiotics. Ceftriaxone was the second most commonly chosen empiric antibiotic overall followed by ciprofloxacin. The response to optimal duration of antibiotic therapy varied widely and highlights the need for national and institutional guidelines.

### Practice

Although 65% of the respondents identified MRSA as the most common resistant organism seen in their practice, it should be noted that at the major referral hospital in the island, MRSA prevalence rate for the past three years has been less than 4% [16]. This information has been widely communicated to local physicians through conferences and workshops. Typing of the MRSA isolates retrieved from this hospital showed that only 29% were scc mec IV type (E. Finlayson, unpub. Data [28]. It should also be noted that 30% and 24% of the MRSA isolates retrieved in 2008 were resistant to low level and high level mupirocin respectively [29] highlighting again the need for constant surveillance.

Similarly, the prevalence rates for VRE have been less than 1% for the past four years in the same hospital (A Nicholson, unpub. data), even though 19% of physicians reported having seen this organism in their practice. As to be expected, hospital physicians reported a higher incidence of MDROs, and PRSPs were more commonly seen in the community.

Although factors influencing choice were appropriate, knowledge of local antibiograms was not always included. Again, this could be related to limited laboratory resources that reduce the availability of culture results and objective data. In the absence of national guidelines, the tendency is for the physicians to use international guidelines, as evidenced by the empiric choices for community and hospital acquired pneumonia and urinary tract infections. This finding also corresponds with the physicians’ high use of the Internet for information (74%), far more than consulting local experts (54%). From the limited susceptibility data available, it is clear that local susceptibility patterns, eg *Staphylococcus aureus* (3.2% MRSA) and *Enterococcus,* (< 1% VRE) (are different from those reported in developed countries, and hence empiric choices should not be solely based on international guidelines. *Streptococcus pneumoniae* is recognized as the commonest cause of community acquired pneumonia and recent data showed a 8% resistance to penicillin (IV) for all isolates nationally (A. Foster, 2015 unpub. data), which is far less than the 24% resistance seen in North America [30]. Where penicillin resistance is low, the use of penicillin/amoxicillin-clavulanic acid for first line therapy is appropriate.

Physicians were more inclined to change to broad-spectrum antibiotics and reluctant to de-escalate, even in the face of laboratory data [31]. This type of practice is expensive, and ultimately drives resistance [32]. In this era of ABR it is important for physicians to achieve optimal outcomes by combining laboratory and clinical data. This study found that only 21% of physicians would de-escalate therapy, while a 2010 single-centre Jamaican study showed that only 7.7% actually practiced de-escalation [33]. There has been some improvement through education of physicians as to the importance of the practice, and attention had been drawn to the results of the 2010 study in conferences, workshops and small group sessions by local microbiologists. Encouragement to de-escalate should continue to be a target in future programmes.

It is encouraging to see that the physicians were paying attention to the need for patient education ranging from explanations of the dangers of unnecessary antibiotics to guiding them to seek follow-up care if necessary. Although 6% admitted to prescribing antibiotics on demand, a further 15% admitted to having done so in the past. This may be the result of increased of continuing education via conferences and workshops, some of which are mandatory. We would have expected more response to patient pressure, especially in the private setting. This is a positive finding, but more work is needed to further reduce this figure.

The majority of physicians were interested in further educational courses on antibiotic resistance. The results of this survey will guide the development of these courses, which could be in the form of hospital based workshops, orientation of new staff and medical conferences.

The major limitation of this study is that it relied on self-reporting by physicians as well as recall of past practices. This could have led to either under or over reporting and recall bias, which may have affected results. Further qualitative research using focus groups, for example, could highlight reasons for some of the practices seen, such as the reluctance to de-escalate therapy, and this could guide interventions.

## Conclusions

Physicians in this study were aware of the problem of ABR, but downplayed its significance nationally and personally. Most had only average knowledge of factors driving resistance, but good knowledge of factors to control resistance. They tended to use international guidelines when making empiric choices, to choose high-powered antibiotics even when patients were improving and to resist de-escalation when appropriate. These are all important targets for a national campaign. Strengthening laboratory medicine in resource limited countries such as Jamaica and most of the English speaking Caribbean countries would play a major role in restricting the emergence of antibiotic resistance. This would also involve training more clinical microbiologists, infectious disease physicians, pharmacists and other experts. This would help to determine prevalent organisms and their antibiograms resulting in more accurate and cost effective therapy. The savings from this could then be invested in an antimicrobial stewardship programme. Policy changes are also needed from local Ministries of Health and national medical associations to provide guidance and monitoring.

## Abbreviations

ABR: Antibiotic resistance
ESBL: Extended spectrum betalactamase
MDROs: Multidrug resistant organisms
MOH: Ministry of Health
MRSA: Methicillin resistant *Staphylococcus aureus*
PRSPs: Penicillin resistant *Streptococcus pneumoniae*
SPSS: Statistical Analysis in the Social Sciences
VRE: Vancomycin resistant Enterococcus
