# Correction to: The knowledge, attitudes and practices of doctors regarding antibiotic resistance at a tertiary care institution in the Caribbean

**DOI:** 10.1186/s13756-018-0358-5

**Published:** 2018-06-25

**Authors:** Alison Nicholson, Ingrid Tennant, Livingston White, Camille-Ann Thoms-Rodriguez, Loraine Cook, Stephen Johnson, Tamara Thompson, Jasper Barnett, Lundie Richards

**Affiliations:** 1 Department of Microbiology, The University of the West Indies (UWI), Kingston, Jamaica; 2 Department of Surgery, Radiology, Anaesthesia and Intensive Care, The University of the West Indies (UWI), Kingston, Jamaica; 3 UWI CARIMAC, Kingston, Jamaica; 4 School of Education, The University of the West Indies (UWI), Kingston, Jamaica; 5 Department of Government, The University of the West Indies (UWI), Kingston, Jamaica; 6 Department of Medicine, The University of the West Indies (UWI), Kingston, Jamaica; 7 Ministry of Health, Kingston, Jamaica

## Correction

The original article [1] contains a major error whereby the present title does not display the correct wording as intended by the authors.

As such, the correct and intended title is as follows, and should instead be taken into account:

“*A national survey of the knowledge, attitudes and prescribing practices of doctors regarding antibiotic resistance in a Caribbean country*”
